# Bidirectional Cause–Effect Relationship Between Urinary Interleukin-6 and Mood, Irritation, and Mental Activity in a Breast Cancer Survivor

**DOI:** 10.3389/fnins.2018.00848

**Published:** 2018-11-28

**Authors:** Christian Schubert, Carmen Hagen

**Affiliations:** ^1^Clinical Department of Medical Psychology, Medical University of Innsbruck, Innsbruck, Austria; ^2^University Hospital Tulln, Tulln an der Donau, Austria

**Keywords:** brain-to-body-to-brain, interleukin-6, emotion, cancer, time series analysis, single-case design

## Abstract

This “integrative single-case study” investigated the bidirectional cause and effect relations between various emotional states (i.e., mood, irritation, mental activity) and urinary IL-6 levels in a 49-year-old female breast cancer survivor (woman) under conditions of “life as it is lived.” During a period of 28 days, the patient collected her entire urine in 12-h intervals for IL-6 measurement and completed each morning and evening a list of adjectives regarding mood, irritation, and mental activity (55 measurements in total). Autoregressive integrated moving average modeling revealed a 4-day (circasemiseptan) cycle in the IL-6 time series. Furthermore, cross-correlational analyses after controlling for serial dependencies (significance level: *p* < 0.05) showed that worsening in mood and increases in irritation were followed by increases in urinary IL-6 levels with temporal delays between 12 and 36 h. In the opposite direction of effect, increases in urinary IL-6 levels were followed by elevations in mood and mental activity as well as decreases in irritation with temporal delays between 48 and 72 h. These results from cross-correlational analyses suggest that IL-6 may have a regulatory function in psychoneuroimmunological interplay and that, under certain conditions, IL-6 may be involved in health rather than sickness behavior. Moreover, the findings of this study are indicators of real-life negative feedback loops and are in line with psychoneuroimmunological research postulating complex brain-to-body-to-brain network-like structures.

## Introduction

Interleukin-6 (IL-6) is a pleiotropic cytokine with pro- and anti-inflammatory properties orchestrating a broad spectrum of immunological and non-immunological reactions (Naugler and Karin, 2008; Scheller et al., 2011; Yirmiya and Goshen, 2011). Under physiological conditions, IL-6 displays regulatory functions in important homeostatic processes of the immune system, the metabolism and the central nervous system. It also represents a keystone cytokine in pathophysiological conditions of a variety of diseases (Erta et al., 2012; Hunter and Jones, 2015; Ghanemi and St-Amand, 2018). In patients with breast cancer, high levels of circulating IL-6 are associated with tumor progression, recurrence, and shorter survival time (Dethlefsen et al., 2013). Furthermore, besides its direct effect on cancer progression, IL-6 is also related to behavioral side effects of breast cancer such as fatigue, depression, and cognitive disturbances (Musselman et al., 2001; Schubert et al., 2007).

As evidence linking IL-6 to poor outcome and low quality of life in breast cancer patients grows, it has become increasingly important to understand how psychosocial factors of daily life affect the synthesis and function of this versatile cytokine. Stress theories consider emotions the final link in the chain from environmental triggers to biological response, making emotions a relevant topic for psychoneuroimmunology (PNI) research (Kemeny, 2007). However, studies on emotions and IL-6 in breast cancer patients are rare, and data have been equivocal. For example, Jehn et al. (2012) found a positive correlation between IL-6 blood levels and depression in a sample of 70 breast cancer patients receiving chemotherapy. Mills et al. (2005), by contrast, found no clear correlation in a sample of 29 breast cancer patients undergoing chemotherapy.

We suggest that these data inconsistency may be due to methodological limitations: Most studies in PNI measure variables at only a few time points and pool data across individuals, thereby ignoring dynamic aspects of the emotion-immune interplay (Rosmalen et al., 2012). We have previously shown in both a healthy female proband and a patient with systemic lupus erythematosus (SLE) that emotionally meaningful incidents and the associated emotional response were followed by cyclic or bi-phasic urinary cortisol and neopterin level changes (Schubert et al., 2003, 2006, 2012). In addition, bi-directional effects, i.e., emotional state affecting IL-6, and, vice versa, IL-6 affecting emotional state (Dantzer et al., 2008), may have been overlooked in conventional nomothetic research designs. We recently found first evidence of real-life, long-term feedback loops between urinary 55 kDa soluble TNF receptor levels and SLE symptoms in mild SLE disease activity (Schubert et al., 2015). Moreover, data averaging studies are often limited in that they choose experimental settings in which the contextual conditions are strongly controlled and restricted in order to increase internal validity. Such approaches, however, ignore the fact that psychological variables are highly dependent on environmental circumstances and should be investigated within the natural ebb and flow of one’s psychosocial reality in order to preserve ecological validity (Van Eck et al., 1996).

We approach these methodological demands on PNI research by applying the “integrative single-case study” design (Schubert et al., 1999, 2012; Singer et al., 2018). Over a period of 55 12-h intervals (28 days), we investigated in the current study the correlations between urinary IL-6 levels and mood, irritation, and mental activity in a patient with prior breast cancer under conditions of “life as it is lived.” As anti-inflammatory medication (Zhang et al., 2017) and physical activity (Meneses-Echávez et al., 2016) are two examples of potential confounders of the IL-6 relations investigated in this study, we included theses variables in the analyses. We used entire urine samples in order to interfere as little as possible with the normal daily routine of the patient and to gather a complete data set without temporal gaps; frequent blood draws would have caused too much sampling stress for the patient and would have yielded gaps in the data set. By applying time series analysis, this study revealed significant effects from emotional states to urinary IL-6 levels and, vice versa, from IL-6 to emotional states.

## Methods

### Study Design

This study is part of a wider project using the “integrative single case study” design (Schubert et al., 1999, 2012) to investigate the impact of emotionally meaningful daily incidents on stress system activity in breast cancer survivors suffering from cancer-related fatigue. Results from this study have already been published (Haberkorn et al., 2013; Singer et al., 2018).

The patient collected her entire urine in intervals of 12 h (from approx. 8:00 p.m. to 8:00 a.m. and from approx. 8:00 a.m. to 8:00 p.m.) for 28 days (from July 13th to August 9th, 2006), froze the aliquoted urine samples at -20°C and brought them to the laboratory once a week, where they were stored at -70°C. Moreover, every 12 h (at approx. 8:00 p.m. and 8:00 a.m.), the patient answered questionnaires regarding emotional state and daily activity, routine, and physical wellbeing. Weekly, the patient was interviewed and assessed medically.

This study was carried out in accordance with the recommendations of the Ethics Review Committee of the University of Freiburg with written informed consent from the subject. The subject gave written informed consent in accordance with the Declaration of Helsinki. The protocol was approved by the Ethics Review Committee of the University of Freiburg.

### Description of the Patient

The patient under study is a 49-year-old Caucasian woman who was diagnosed with a ductal mamma carcinoma (C50.4) at stage pT2 [pN1biv (6 of 13), cM0, G3, R0, ER 10%, PR 70–80%, HER2+/neu+, score = 3] in her right breast 5 years before the study started. She was treated with surgery, radiotherapy, and anti-estrogen therapy (tamoxifen), the latter ending 6 months before study start. Physical examination prior to study start revealed that she was free of tumor and metastatic lesions or recurrence. Psychological examination showed that the patient was suffering from chronic depressive symptoms (F34.1) that had increased markedly (adjustment disorder with depressed mood [F43.21]) after her cancer diagnosis. She also developed severe cancer-related fatigue following her diagnosis. She saw a psychotherapist for 6 months, ending 5 months before study start.

The depression and fatigue comorbidity at study start was objectified through high scores on the Center for Epidemiological Studies Depression Scale (CES-D, 50 out of 60) and the “general fatigue” subscale of the Multidimensional Fatigue Inventory-20 (MFI-20, 20 out of 20). The patient has German “Abitur” qualification (high school diploma with university entrance examination) and is a physiotherapist; she is married to a doctor specialized in psychosomatic medicine and psychotherapy; they have three children. The socioeconomic status of the patient may be considered upper middle class according to the classification of the German Socio-Economic Panel (SOEP) (Niehues, 2017). She consumes alcohol moderately and is a non-smoker. At study start, the only medication the patient took was aspirin^®^ (acetyl-salicylic acid, 500 mg) on an irregular basis.

### Measurement of Emotional States

To measure emotional states, we used the short form of the 3-Skalen-Eigenschaftswörterliste (3-Skalen-EWL), a self-report paper and pencil test with high internal consistency (Becker, 1988). The 3-Skalen-EWL consists of 28 adjectives assigned to three emotional categories, i.e., mood, irritation, and mental activity. Each adjective was rated by the patient on a four-point Likert Scale (1 = not at all, 2 = a little, 3 = quite, 4 = very). The questionnaire takes only a few minutes to complete. By averaging the single scores, we calculated a summary score between 1 and 4 for each emotional category.

### Measurement of Physical Activity

For 12-h measurement of physical activity, a visual analog scale (VAS) was used, consisting of a 10 cm-long horizontal line headed by “physical strain during the day” or “physical strain since yesterday evening.” One end of the line is marked with “not at all,” the other with “very.” The patient placed a mark on this line to indicate the intensity of her physical activity.

### Measurement of Interleukin-6 per Creatinine

This study used urine for the determination of IL-6. Nowak et al. (2012) have shown an IL-6 pass ratio of 0.2% from renal arteries and veins to urine indicating the direct proportional quantity of serum IL-6 excreted in urine.

After thawing from -70°C, the 55 consecutive urinary IL-6 concentrations were measured in one single run. The delay between urine collection/freezing and thawing ranged from approx. 6 months (for creatinine) to approx. 2 years (for IL-6). Urine samples were never frozen and thawed more than once.

Urinary IL-6 levels were determined through ELISA, as recommended by the manufacturer (Human IL-6 Quantikine^®^ HS ELISA, R&D Systems, Minneapolis, MN). The Quantikine^®^ HS ELISA has a coefficient of variation for urine determination of 5.5–9.8% for intra-assay precision and 5.5–11.2% for inter-assay precision. The normal range of IL-6 in urine is from not determinable to 6.76 pg/ml. Urinary IL-6 analyses were performed in singulate.

For creatinine measurement, high pressure liquid chromatography (HPLC) (Model ProStar 210 Solvent Delivery Modul; Varian Associates, Palo Alto, CA) was applied as described (Fuchs et al., 1992). Urinary creatinine levels were measured five times. For each of the five independent creatinine determinations, a new aliquot was used, and results were averaged.

Urinary IL-6 concentrations were expressed in milligram per mole (mg/mol) creatinine to compensate for urine density.

### Statistical Analyses

Cross-correlational analyses between IL-6-levels and the other variables under investigation were performed at lag 0 and at higher lags up to ±7 using SPSS-Trends^TM^ 24.0 (IBM Corp., 2016). We controlled for spurious cross-correlations due to trends (e.g., circadian rhythm) and serial dependencies (e.g., autoregression) by cross-correlating residual series after autoregressive integrated moving average (ARIMA) modeling of the time series (Box and Jenkins, 1976). When the mean of a series needed to be stabilized, the series was detrended either by removing a linear trend or by differencing. Transformation of a series (e.g., log, square root) was applied in order to stabilize variance, to correct abnormal distribution, and to improve model specification. The final models were chosen depending on the statistical significance of model parameters, the information criteria (e.g., root-mean-square deviation [RMSE], normalized Bayesian information criterion [BIC]), and the serial independency of residuals indicated by the Ljung-Box test (Box and Jenkins, 1976). A missing value in a time series was corrected by linear interpolation. The level α of statistical significance was *p* < 0.05. Exact *p* values were determined through Gaussian approximation.

## Results

Time series analysis was based on 55 measurements covering a period of 28 days or 55 12-h intervals. No signs of infection were observed during the study period.

The time series of mood had one missing value at 12-h unit 52, while all other time series were complete. The mean levels were as follows: IL-6 (pg/ml): 0.6 ± 0.6 (range 0.2–2.6); creatinine (μmol/l): 5.6 ± 2.5 (range 2.6–13.3); IL-6/creatinine (mg/mol): 0.1 ± 0.1 (range 0.02–0.5); mood: 2.2 ± 0.6 (range 1.0–3.6); irritation 2.4 ± 0.6 (range 1.6–3.4); mental activity 1.5 ± 0.3 (range 1.0–2.0); physical activity 2.2 ± 2.6 (range 0.0–8.5). The following ARIMA models were found: IL-6 SAR(1), *s* = 8; mood AR(2); irritation MA(1); mental activity AR(4,1) ln; physical activity MA(2) SAR(1), *s* = 10, ln.

**FIGURE 1:**
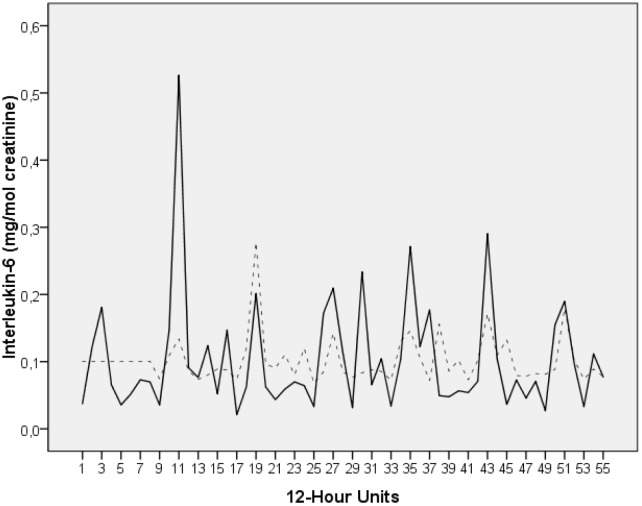
Time series of urinary IL-6 levels of the breast cancer patient under study (milligram per mole creatinine). The time series covers a period of 28 days. During this time interval, the patient collected her entire urine in 12-h intervals, resulting in a total of 55 measurements (day portions from 8:00 a.m. to 8:00 p.m. and night portions from 8:00 p.m. to 8:00 a.m.). The raw data (solid black) and the fit from modeling the series (dotted grey) are plotted. The time series starts with a night portion.

Figure 1 shows the time series for IL-6 (mg/mol creatinine).

**FIGURE 2:**
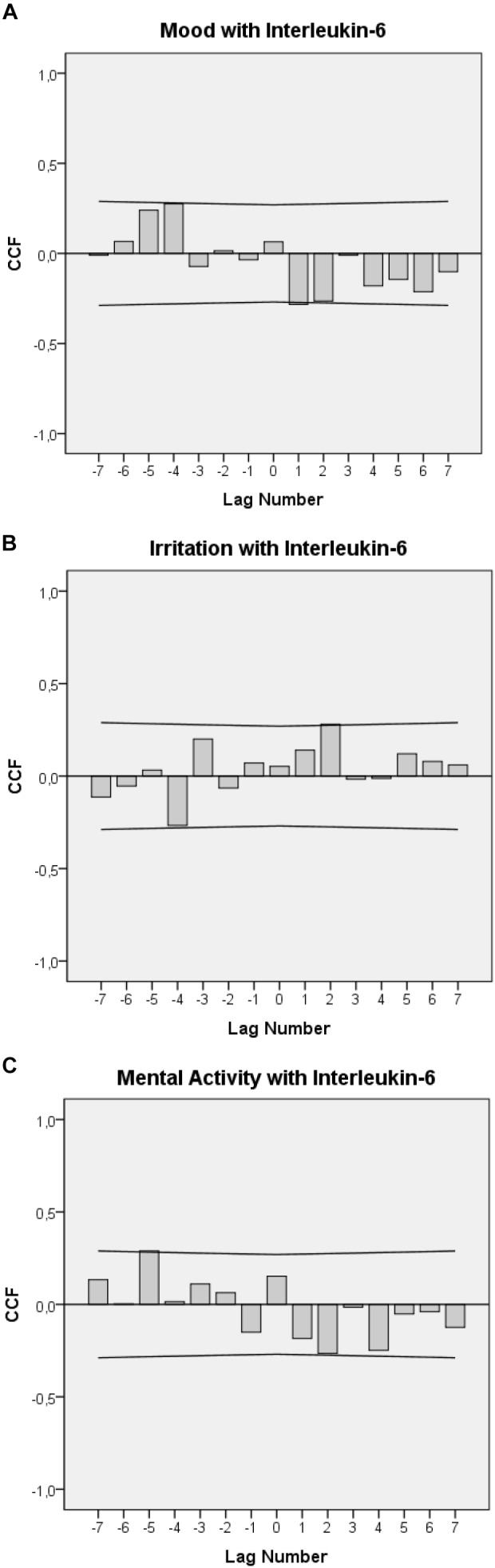
Cross-correlation functions (CCF) between urinary IL-6 levels, mood **(A)**, irritation **(B)**, and mental activity **(C)**. Each lag represents a 12-h interval. Plots show cross-correlation coefficients (bars) and upper and lower limits of the 95% confidence intervals. Significance level is *p* < 0.05. A positive lag significance means that levels of mood, irritation, and mental activity precede IL-6 levels. A negative lag significance means that IL-6 levels precede levels of mood, irritation, and mental activity. The change in the sign of the cross-correlation functions between positive and negative lags indicates negative feedback loops **(A–C)**.

Figures 2A–C display the cross-correlational functions between IL-6 levels and each of the emotional state variables. Figure 2A shows that a worsening in mood was followed by a significant increase in urinary IL-6 concentrations with temporal delays of 12–24 h (lag 1: *r* = -0.284, *p* = 0.037) and, tendentially, of 24–36 h (lag 2: *r* = -0.265, n.s.). In addition, in the opposite direction of effect, increases in urinary IL-6 levels were followed by significant elevations in mood by 48–60 h (-lag 4: *r* = +0.275, *p* = 0.049).

Figure 2B shows that an increase in irritation was followed by a significant increase in urinary IL-6 concentrations with a delay of 24–36 h (lag 2: *r* = –0.281, *p* = 0.041) and that, in the opposite direction, increases in urinary IL-6 levels were followed by decreases in irritation with a delay of 48–60 h (-lag 4: *r* = -0.284, *p* = 0.043).

Figure 2C shows that a decrease in mental activity was tendentially followed by increases in urinary IL-6 concentrations with a delay of 24–36 h (lag 2: *r* = -0.265, n.s.) and that increases in urinary IL-6 levels were followed by significant increases in mental activity with a delay of 60–72 h (-lag 5: *r* = +0.289, *p* = 0.041).

The patient took aspirin^®^ on five occasions during the study period – specifically on 12-h units 2 (1 mg × 500 mg), 26 (3 mg × 500 mg), 27 (2 mg × 500 mg), 28 (2 mg × 500 mg), 34 (2 mg × 500 mg). Aspirin^®^ intake was not significantly cross-correlated with urinary IL-6 levels in either direction of effect (data not shown). Similarly, changes in physical activity were not significantly cross-correlated with urinary IL-6 levels (data not shown).

## Discussion

This “integrative single case study” on a 49-year-old breast cancer survivor provided first insights into the bidirectional psycho-immunological dynamics of IL-6 under conditions of “life as it is lived.” Univariate ARIMA modeling of the urinary IL-6 time series revealed a stochastic seasonal first-order autoregressive process (SAR [1]) with a span of seasonality of 8, i.e., 4 days. This suggests a roughly half-weekly (circasemiseptan) rhythm in the IL-6 chronome of the patient. Circasemiseptan rhythms in cancer patients have also been found in other biological processes such as mitotic activity and heart rate variability (Blank et al., 1999).

Cross-correlational analyses revealed significant correlations in both directions of effect in the patient’s emotion-immune interplay. In one direction of effect, a worsening in mood and an increase in irritation were followed by significant increases in urinary IL-6 levels 12–24 h and 24–36 h later, respectively. Moreover, 24–36 h after a decrease in mental activity, a tendency toward an increase in IL-6 levels was observed. These findings demonstrate a clear impact of emotional state on urinary IL-6 levels under naturalistic conditions, thus rendering IL-6 a possible biological mediator through which everyday life may influence tumor progression and survival in the breast cancer patient under study (Dethlefsen et al., 2013).

Looking at the opposite direction of effect, increases in urinary IL-6 levels significantly preceded elevations in mood and mental activity by 48–60 h and 60–72 h, respectively, and decreases in irritation by 48–60 h. These rather long temporal delays between immune activity and changes in emotional states may be based on multisite cascades mediating between immune system and central nervous system activities, which are functionally linked via complex regulation loops (Besedovsky and del Rey, 2011; Yirmiya and Goshen, 2011).

Indeed, the cross-correlational functions seen in Figures 2A–C suggest not only clear linear connections between emotions and immune activity in the patient under study but further indicate that emotional states and IL-6 may be part of a complex functional circuit. The cross-correlational constellations seen in Figures 2A–C, in which a negative (positive) value in one process becomes a positive (negative) value after having interacted with the other process, and vice versa, are an indicator of a negative feedback loop (von Storch, 2000; Schubert et al., 2015). From this perspective, the cross-correlogram in Figure 2A could be read as follows: Worsened mood may have initially raised IL-6 levels after 12–24 h (positive lag and negative correlation in Figure 2A). These raised IL-6 levels may have then, in the manner of a feedback mechanism, led to elevated mood after a lag of 60–72 h (negative lag and positive correlation in Figure 2A). In this patient, similar feedback loops may also exist between IL-6 and irritation (Figure 2B) and IL-6 and mental activity (Figure 2C).

A great advantage of the research design applied in this study is that it enables us, by taking a step back from fragmented entities, to look at the broader picture of the dynamic interplay of real-life biological, psychological, and social data. Importantly, the negative feedback loops we found between emotional states and IL-6 activities under conditions of “life as it is lived” fit into network-like conceptualizations taken from experimental evidence. Besedovsky and del Rey (2011), for example, postulate a complex circuit between central and peripheral neuro-immune activities. In their view, central immune activity – involved in, for example, memory processes – triggers central neuro-hormonal responses (e.g., hypothalamic–pituitary–adrenal axis, autonomic nervous system) that can alter peripheral immune parameters that in turn influence central immune responses, creating a brain-to-body-to-brain reverberating feedback loop. IL-6, being able to pass the blood–brain barrier via specific transports (Banks et al., 1995), is thought to be a key endocrine mediator in this neuroimmune circuit.

The role of IL-6 in this circuit may be quite complex and may influence CNS functioning in different and even opposite ways under various conditions (Yirmiya and Goshen, 2011). Specifically, while markedly elevated IL-6 levels in connection with pro-inflammatory activity can have detrimental effects on cognitive functions such as memory, basal IL-6 under physiological conditions plays an important role in the regulation of neuronal and synaptic functions, including synaptic transmission and plasticity (Besedovsky and del Rey, 2011; Yirmiya and Goshen, 2011; Gruol et al., 2014).

The patient of this study had normal urinary IL-6 values despite ongoing depression and fatigue, both of which are usually associated with enhanced IL-6 levels in breast cancer survivors (Musselman et al., 2001; Schubert et al., 2007). Thus, by demonstrating that increases in the patient’s urinary IL-6 levels were followed by elevations of positive emotional states, our findings might reflect normal physiological processes. Moreover, the psychologically regenerative function of IL-6 observed in this study suggests that IL-6 can be anti-inflammatory, promoting a behavior that is more related to health than to sickness (Scheller et al., 2011; Dethlefsen et al., 2013). This appears reasonable as a system that can give rise to sickness behavior to protect the individual in case of disease (Dantzer et al., 2008) may also ensure the reestablishment of health by stimulating positive health-related behavioral and social activities (Eisenberger et al., 2016). Further evidence for such “health behavior” is seen in a study on healthy individuals where those participants who showed greater increases in circulating IL-6 in response to endotoxin also showed greater activity in the ventral striatum – a key reward-related neural region – in response to viewing images of loved ones (Inagaki et al., 2015). Moreover, even in populations with inflammatory background, such as chronic fatigue syndrome and SLE, IL-6 was found to have protective rather than disturbing effects on cognition (Kozora et al., 2001; Arnold et al., 2002).

Previous evaluations of the integrative single-case study presented here focused on the bidirectional effects between neopterin and various other variables associated with the patient’s depression and fatigue comorbidity. Neopterin is a marker of enhanced cellular immune activation and oxidative stress that has been associated with poorer health outcomes and lower survival rates in breast cancer (Murr et al., 1999). In the patient of this study, we found that increases in urinary neopterin concentrations preceded increases in fatigue intensity with a temporal delay of 60–72 h, whereas elevations of the patient’s mood were followed by a cyclic response pattern of urinary neopterin levels with an ultimate decrease in neopterin after 132–144 h (Haberkorn et al., 2013). In addition, increases in the patient’s positive sleep variables (i.e., sleep quality, sleep recreational value) were followed by urinary neopterin concentration decreases after 96–120 h and 120–144 h. Conversely, increases in total wake time, a negative sleep variable, were followed by increases in urinary neopterin levels 72–96 h later (Singer et al., 2018). These findings from previous evaluations show that not only the patient’s urinary IL-6 but also her urinary neopterin concentrations were dynamically interrelated with various emotional and behavioral variables.

A strength but also a limitation of this integrative single-case study is its exploratory nature. It remains unclear whether our observations are typical only for the depressed and fatigued breast cancer survivor under study or for most other cancer patients, or can even be applied to healthy individuals. Thus, further investigation and replication of findings are needed for generalization. With specific regard to the findings on the emotion-IL-6 interplay seen in this study, other biomarkers (e.g., epinephrine, cortisol) that may similarly orchestrate “health behavior” should be addressed in future evaluations. Moreover, as 12-h self-reported measures of aspirin^®^ intake and physical activity did not correlate with urinary IL-6 levels, other factors known for their influence on the emotion-IL-6 interplay, such as dietary intake (Kiecolt-Glaser, 2010), should be integrated in further work as well. Finally, the statistical tools in our project could be expanded to include multivariate statistics (e.g., vector autoregressive [VAR] modeling, impulse-response function [IRF] analysis) specified to test the occurrence of feedback mechanisms in time series data (Lütkepohl, 2006).

## Conclusion

In conclusion, this integrative single-case study on a breast cancer survivor suffering from depression and fatigue showed that positive emotional states such as elevated mood can be both positively and negatively correlated with IL-6 – in both directions of effect, with different temporal delays and intertwined with feedback circuits. Such findings confirm recent data from our working group on the complexity of emotion–immune interplay under conditions of “life as it is lived” (Schubert et al., 2003, 2006, 2012, 2015; Haberkorn et al., 2013; Singer et al., 2018). The results of this study again raise the possibility that data inconsistency in current PNI research may result from not taking the complex functional aspects of life-science data into account.

## Author Contributions

CS and CH did substantial contributions to the conception or design of the work, the acquisition, analysis, or interpretation of data for the work. CH and CS drafted the work or revised it critically for important intellectual content. CS and CH did final approval of the version to be published. CS and CH did agreement to be accountable for all aspects of the work in ensuring that questions related to the accuracy or integrity of any part of the work are appropriately investigated and resolved.

## Conflict of Interest Statement

The authors declare that the research was conducted in the absence of any commercial or financial relationships that could be construed as a potential conflict of interest.

## References

[B1] Arnold M. C. Papanicolaou D. A. O’Grady J. A. Lotsikas A. Dale J. K. Straus S. E. (2002). Using an interleukin-6 challenge to evaluate neuropsychological performance in chronic fatigue syndrome. *Psychol. Med.* 32 1075–1089. 10.1017/S0033291702006086 12214788

[B2] Banks W. A. Kastin A. J. Broadwell R. D. (1995). Passage of cytokines across the blood-brain barrier. *Neuroimmunomodulation* 2 241–248. 10.1159/000097202 8963753

[B3] Becker P. (1988). Skalen für verlaufsstudien der emotionalen Befindlichkeit. *Zschr. Exp. Angew. Psychol.* 3 345–369.

[B4] Besedovsky H. O. del Rey A. (2011). Central and peripheral cytokines mediate immune-brain connectivity. *Neurochem. Res.* 36 1–6. 10.1007/s11064-010-0252-x 20820913

[B5] Blank M. Denisova O. Cornélissen G. Halberg F. (1999). Enhanced circasemiseptan (about 3.5-day) variation in the heart rate of cancer patients?. *Anticancer Res.* 19 853–855. 10216505

[B6] Box G. E. P. Jenkins G. M. (1976). *Time Series Analysis: Forecasting and Control.* San Francisco, CA: Holden-Day.

[B7] Dantzer R. O’Connor J. C. Freund G. G. Johnson R. W. Kelley K. W. (2008). From inflammation to sickness and depression: when the immune system subjugates the brain. *Nat. Rev. Neurosci.* 9 46–56. 10.1038/nrn2297 18073775 PMC2919277

[B8] Dethlefsen C. Højfeldt G. Hojman P. (2013). The role of intratumoral and systemic IL-6 in breast cancer. *Breast Cancer Res. Treat.* 138 657–664. 10.1007/s10549-013-2488-z 23532539

[B9] Eisenberger N. I. Moieni M. Inagaki T. K. Muscatell K. A. Irwin M. R. (2016). In sickness and in health: the co-regulation of inflammation and social behavior. *Neuropsychopharmacology* 42 242–253. 10.1038/npp.2016.141 27480575 PMC5143485

[B10] Erta M. Quintana A. Hidalgo J. (2012). Interleukin-6, a major cytokine in the central nervous system. *Int. J. Biol. Sci.* 8 1254–1266. 10.7150/ijbs.4679 23136554 PMC3491449

[B11] Fuchs D. Weiss G. Reibnegger G. Wachter H. (1992). The role of neopterin as a monitor of cellular immune activation in transplantation, inflammatory, infectious, and malignant diseases. *Crit. Rev. Clin. Lab. Sci.* 29 307–341. 10.3109/10408369209114604 1489521

[B12] Ghanemi A. St-Amand J. (2018). Interleukin-6 as a “metabolic hormone”. *Cytokine* 5 S1043–S4666. 10.1016/j.cyto.2018.06.034 29983356

[B13] Gruol D. L. Vo K. Bray J. G. (2014). Increased astrocyte expression of IL-6 or CCL2 in transgenic mice alters levels of hippocampal and cerebellar proteins. *Front. Cell. Neurosci.* 8:234 10.3389/fncel.2014.00234 PMC4132577 25177271

[B14] Haberkorn J. Burbaum C. Fritzsche K. Geser W. Fuchs D. Ocaña-Peinado F. M. (2013). Day-to-day cause-effect relations between cellular immune activity, fatigue and mood in a patient with prior breast cancer and current cancer-related fatigue and depression. *Psychoneuroendocrinology* 38 2366–2372. 10.1016/j.psyneuen.2013.03.001 23541233

[B15] Hunter C. A. Jones S. A. (2015). IL-6 as a keystone cytokine in health and disease. *Nat. Immunol.* 16 448–457. 10.1038/ni.3153 25898198

[B16] IBM Corp. (2016). *IBM SPSS Statistics for Windows, Version 24.0.* Armonk, NY: IBM Corp.

[B17] Inagaki T. K. Muscatell K. A. Irwin M. R. Moieni M. Dutcher J. M. Jevtic I. (2015). The role of the ventral striatum in inflammatory-induced approach toward support figures. *Brain Behav. Immun.* 44 247–252. 10.1016/j.bbi.2014.10.006 25459101 PMC4275369

[B18] Jehn C. F. Flath B. Strux A. Krebs M. Possinger K. Pezzutto A. (2012). Influence of age, performance status, cancer activity, and IL-6 on anxiety and depression in patients with metastatic breast cancer. *Breast Cancer Res. Treat.* 136 789–794. 10.1007/s10549-012-2311-2312 23124416

[B19] Kemeny M. E. (2007). “Emotions and the immune system,” in *Psychoneuroimmunology* 4th Edn ed. Ader R. (New York, NY: Elsevier) 619–629. 10.1016/B978-012088576-3/50035-6

[B20] Kiecolt-Glaser J. K. (2010). Stress, food, and inflammation: psychoneuroimmunology and nutrition at the cutting edge. *Psychosom. Med.* 72 365–369. 10.1097/PSY.0b013e3181dbf489 20410248 PMC2868080

[B21] Kozora E. Laudenslager M. Lemieux A. West S. G. (2001). Inflammatory and hormonal measures predict neuropsychological functioning in systemic lupus erythematosus and rheumatoid arthritis patients. *J. Int. Neuropsychol. Soc.* 7 745–754. 10.1017/S1355617701766106 11575596

[B22] Lütkepohl H. (2006). *New Introduction to Multiple Time Series Analysis 1st edn.* Berlin: Springer.

[B23] Meneses-Echávez J. F. Correa-Bautista J. E. González-Jiménez E. Schmidt Río-Valle J. Elkins M. R. Lobelo F. (2016). The effect of exercise training on mediators of inflammation in breast cancer survivors: a systematic review with meta-analysis. *Cancer Epidemiol. Biomark. Prev.* 25 1009–1017. 10.1158/1055-9965.EPI-15-1061 27197276

[B24] Mills P. J. Parker B. Dimsdale J. E. Sadler G. R. Ancoli-Israel S. (2005). The relationship between fatigue and quality of life and inflammation during anthracycline-based chemotherapy in breast cancer. *Biol. Psychol.* 69 85–96. 10.1016/j.biopsycho.2004.11.007 15740827

[B25] Murr C. Fuith L. C. Widner B. Wirleitner B. Baier-Bitterlich G. Fuchs D. (1999). Increased neopterin concentrations in patients with cancer: indicator of oxidative stress? *Anticancer Res.* 19 1721–1728. 10470106

[B26] Musselman D. L. Miller A. H. Porter M. R. Manatunga A. Gao F. Penna S. (2001). Higher than normal plasma interleukin-6 concentrations in cancer patients with depression: preliminary findings. *Am. J. Psychiatry* 158 1252–1257. 10.1176/appi.ajp.158.8.1252 11481159

[B27] Naugler W. E. Karin M. (2008). The wolf in sheep’s clothing: the role of interleukin-6 in immunity, inflammation and cancer. *Trends Mol. Med.* 14 109–119. 10.1016/j.molmed.2007.12.007 18261959

[B28] Niehues J. (2017). Die mittelschicht in deutschland: vielschichtig und stabil. *Vierteljahresschrift empirischen Wirtschaftsforschung* 44 3–20. 10.2373/1864-810X.17-01-01

[B29] Nowak M. Wyczalkowska-Tomasik A. Wlodarczyk Z. Paczek L. (2012). The role of the kidney in the systemic elimination of interleukin 6, platelet-derived growth factor and transforming growth factor beta. *Cytokine* 59 258–263. 10.1016/j.cyto.2012.04.041 22617683

[B30] Rosmalen J. G. Wenting A. M. Roest A. M. de Jonge P. Bos E. H. (2012). Revealing causal heterogeneity using time series analysis of ambulatory assessments: application to the association between depression and physical activity after myocardial infarction. *Psychosom. Med.* 74 377–386. 10.1097/PSY.0b013e3182545d47 22582335

[B31] Scheller J. Chalaris A. Schmidt-Arras D. Rose-John S. (2011). The pro- and anti-inflammatory properties of the cytokine interleukin-6. *Biochim. Biophys. Acta* 1813 878–888. 10.1016/j.bbamcr.2011.01.034 21296109

[B32] Schubert C. Geser W. Noisternig B. Fuchs D. Welzenbach N. König P. (2012). Stress system dynamics during “life as it is lived”: an integrative single-case study on a healthy woman. *PLoS One* 7:e29415. 10.1371/journal.pone.0029415 22403606 PMC3293932

[B33] Schubert C. Haberkorn J. Ocaña-Peinado F. M. König P. Sepp N. Schnapka-Köpf M. (2015). Cause-effect relations between 55 kD soluble TNF receptor concentrations and specific and unspecific symptoms in a patient with mild SLE disease activity: an exploratory time series analysis study. *BMC Res. Notes* 8:465. 10.1186/s13104-015-1398-z 26391351 PMC4578846

[B34] Schubert C. Hong S. Natarajan L. Mills P. J. Dimsdale J. E. (2007). The association between fatigue and inflammatory marker levels in cancer patients: a quantitative review. *Brain Behav. Immun.* 21 413–427. 10.1016/j.bbi.2006.11.004 17178209

[B35] Schubert C. Lampe A. Geser W. Noisternig B. Fuchs D. König P. (2003). Daily psychosocial stressors and cyclic response patterns in urine cortisol and neopterin in a patient with systemic lupus erythematosus. *Psychoneuroendocrinology* 28 459–473. 10.1016/S0306-4530(02)00034-3 12573308

[B36] Schubert C. Lampe A. Rumpold G. Fuchs D. König P. Chamson E. (1999). Daily psychosocial stressors interfere with the dynamics of urine neopterin in a patient with systemic lupus erythematosus: an integrative single-case study. *Psychosom. Med.* 61 876–882. 10.1097/00006842-199911000-00024 10593641

[B37] Schubert C. Noisternig B. Fuchs D. König P. Chamson E. Mittnik S. (2006). Multifaceted effects of positive incidents on urine cortisol and urine neopterin dynamics in a patient with systemic lupus erythematosus. *Stress Health* 22 215–227. 10.1002/smi.1096

[B38] Singer M. Burbaum C. Fritzsche K. Peterlini S. Bliem H. R. Ocaña-Peinado F. M. (2018). Subjective positive and negative sleep variables differentially affect cellular immune activity in a breast cancer survivor: a time-series analysis approach. *Front. Neurol.* 8:693. 10.3389/fneur.2017.00693 29375463 PMC5767176

[B39] Van Eck M. M. Nicolson N. A. Berkhof H. Sulon J. (1996). Individual differences in cortisol responses to a laboratory speech task and their relationship to responses to stressful daily events. *Biol. Psychol.* 43 69–84. 10.1016/0301-0511(95)05159-7 8739615

[B40] von Storch J.-S. (2000). Signatures of air–sea interactions in a coupled atmosphere–ocean GCM. *J. Clim.* 13 3361–3379.

[B41] Yirmiya R. Goshen I. (2011). Immune modulation of learning, memory, neural plasticity and neurogenesis. *Brain Behav. Immun.* 25 181–213. 10.1016/j.bbi.2010.10.015 20970492

[B42] Zhang Y. Kong W. Jiang J. (2017). Prevention and treatment of cancer targeting chronic inflammation: research progress, potential agents, clinical studies and mechanisms. *Sci. China Life Sci.* 60 601–616. 10.1007/s11427-017-9047-9044 28639101

